# Spontaneous relational and object similarity in wild bumblebees

**DOI:** 10.1098/rsbl.2022.0253

**Published:** 2022-08-31

**Authors:** Gema Martin-Ordas

**Affiliations:** ^1^ Division of Psychology, University of Stirling, Stirling FK9 4LA, UK; ^2^ Department of Psychology, University of Oviedo, 33003 Asturias, Spain

**Keywords:** relational similarity, object similarity, reasoning, invertebrates, bumblebees

## Abstract

Being able to abstract relations of similarity is considered one of the hallmarks of human cognition. While previous research has shown that other animals (e.g. primates) can attend to relational similarity, they struggle to focus on object similarity. This is in contrast with humans. And it is precisely the ability to attend to objects that it is argued to make relational reasoning uniquely human. What about invertebrates? Despite earlier studies indicating that bees are capable of learning abstract relationships (e.g. ‘same’ and ‘different’), no research has investigated whether bees can spontaneously attend to relational similarity and whether they can do so when relational matches compete with object matches. To test this, a spatial matching task (with and without competing object matches) previously used with children and great apes was adapted for use with wild-caught bumblebees. When object matches were not present, bumblebees spontaneously used relational similarity. Importantly, when competing object matches were present, bumblebees still focused on relations over objects. These findings indicate that the absence of object bias is also present in invertebrates and suggest that the relational gap between humans and other animals is due to their preference for relations over objects.

## 1. Introduction

A common mistake that preschool—but not older—children make when asked ‘duck is to duckling as tiger to?’ is to answer ‘duckling’ (i.e. *object/perceptual similarity*) instead of ‘cub’ (i.e. *relational similarity*) [1]. Understanding that critical object properties are not necessarily the properties of the objects individually, but the relations of the properties of the different objects to each other is fundamental for recognizing relational similarity [2]. The capacity to perceive relational similarity as different from object similarity impacts reasoning and is often considered a hallmark of human cognition [3].

Using match-to-sample tasks (MTS)—in which a sample stimulus is presented with two comparison stimuli, a correct and an incorrect match—vertebrates (e.g. primates and birds [1,4]) and invertebrates (e.g. bees [5]) have been shown to recognize relational similarity. However, there are shortcomings with this paradigm. The MTS entails a large amount of training and performance can be explained by simply accounting for the perceptual variability between the presented stimuli—rather than by identifying the relation between the stimuli [6]. Whereas work with vertebrates has overcome these issues by using spatial mapping tasks (e.g. [1,7]), it is still unknown if invertebrates could recognize relational similarity in these tasks.

Most of the evidence on relational processing (i.e. acquiring and extrapolating implicit knowledge) and relational learning (e.g. learning same/different, larger/smaller rules) comes from honeybees (e.g. [5,8–10]); however, bumblebees can also learn to process relations [11] and solve problems never before encountered in their evolutionary history [12,13]. Additionally, bumblebees have extraordinary spatial memory skills (e.g. [14,15]). As such, they are good candidates to investigate their relational skills in a spatial mapping task. For this purpose, a spatial mapping paradigm developed by Christie *et al*. [1] was modified for use with wild-caught bees. Specifically, I examined bumblebees' relational reasoning in two tasks: (i) a pure relational task (Experiment 1) and (ii) a relational task that included competing object matches (Experiment 2). Previous research in bees has not investigated *spontaneous* relational reasoning when relational matches compete with object matches. Addressing this issue is important because establishing comparisons between similar objects (e.g. bicycles and tricycles are vehicles) contributes to develop abstract relationships (e.g. skateboards are also vehicles) and, so far, only humans have been shown to do so [16]. Thus, it is expected that, like in other animals, bees recognize relational similarities spontaneously and show a preference for relational matches compared to concrete objects.

## 2. Experiment 1: relational similarity

Bumblebees experienced two sets of objects (baited and searching arrays). First, they were presented with two baited objects—only one them was dipped in sucrose. Their task was to find the corresponding strip in the searching array. This experiment examined whether bees would spontaneously use relational alignment in their searches. There were two conditions: *aligned* and *misaligned*. In the *aligned* condition, the baited and searching arrays were spatially aligned: top strip- > top strip, bottom strip- > bottom strip. In the *misaligned* condition, only the bottom strip of the baited array was aligned with the top strip of the searching array. It was predicted that if bees establish a relational correspondence between the baited and searching arrays, they should search top- > top, bottom- > bottom regardless of the alignment of strips. However, if bees consider each strip independently, they would struggle with the bottom trials in the *misaligned* condition. Consequently, a worse performance in the *misaligned* condition compared to the *aligned* condition would be predicted.

### (a) Methods

#### (i) Subjects

The data were collected between August and September 2021 in Northumberland (UK). A total of 33 bees were captured, although three of them completed less than six trials and were not included in the analyses. The final sample was 30 bees of the following species: *Bombus pascuorum* (*n* = 23), *Bombus lapidarious* (*n* = 3), *Bombus lucorum* (*n* = 2), *Bombus terrestris* (*n* = 1) and *Bombus bohemicus* (*n* = 1). Sex was visually identified (females = 11; males = 11 and eight could not be clearly identified).

#### (ii) Apparatus

**Figure 1.**
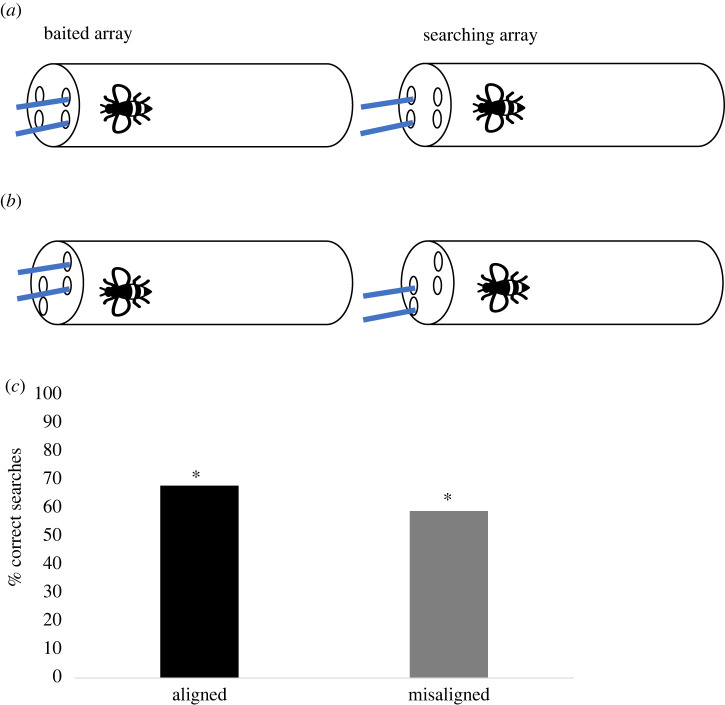
Experimental set-up of Experiment 1. Bees experience one of the two paper strips baited on the (i) (E's perspective; baited array), and they had to search among the containers on the (ii) (searching array). All the stimuli were identical. In both the aligned (*a*) and misaligned (*b*) conditions, the correct mapping between hide and search is the parallel spatial relations. Importantly, in the misaligned condition (*b*), the bottom strip of the baited array spatially matches the top strip of the search array. If bees perceive relational similarity, they should perform better in the aligned than in the misaligned condition; (*c*) represents the percentage of correct responses in the aligned and misaligned condition (Experiment 1). Bees searched in the correct location significantly above chance in both conditions. *p < 0.05.

A transparent plastic tube (14 × 3.5 cm) with four holes at the end through which the stimuli could be inserted was used. Two holes were drilled on the right-hand side of the tube and the other two holes on the left-hand side of the tube (figure 1*a,b*). For both conditions, the distance between the top right hole and the top and bottom left holes was equal (2 cm). The same was true for the bottom left hole. This allowed to control for proximity as a potential factor influencing bees’ searches. Blue strips of paper (3 × 0.2 cm) were used as stimuli: two were introduced through the right side of the tube (baited array) and two through the left side (searching array). The strips were fixed in Playdoh to introduce them simultaneously in the tube.

#### (iii) Procedure

I followed Muth *et al*.'s [17] procedure to test wild-caught bees. Subjects were left in the tube on average for 2 h prior to testing to allow them to habituate to the tubes and become motivated to forage.

The procedure was the same for the *aligned* and *misaligned* conditions (figure 1*a,b*). Subjects first were presented with the baited strips on the right-hand side of the tube (Experimenter's (E) perspective). Only one of the strips—either top or bottom—was dipped in 50% (w/w) sucrose. Bees were allowed to explore both strips so they could notice that only one of them was rewarded. Once the bee made contact with the strip dipped in sucrose—either by using its antennae or proboscis—it was given (on average) 5–6 s to drink the solution. Then, the baited objects were removed and the E introduced the searching strips. These strips were dipped in water. A choice was considered when the bees touched the strip with the antennae or proboscis. Each bee received a total of 12 trials and the position of the reward—top or bottom—was counterbalanced across trials. New paper strips were used for each trial and in each array. Importantly, bees did not receive any training prior to these trials and their choices were never rewarded.

#### (iv) Analyses

Data were analysed using R v.2022.02.0 using a binomial general liner mixed model [18]. The dependent variable was whether bees' choice was correct (coded 1) or incorrect (coded 0), the independent variable was experimental condition as a categorical variable and a random factor was the individual bees. In the *aligned* and *misaligned* conditions, spatial matches were considered as correct. Wilcoxon tests were used to analyse if performance in each condition was significantly above chance.

### (b) Results and discussion

There was no effect of condition on bees’ performance (estimate s.d. = −0.384, *z* = 1.773, *p* = 0.060, 95% CI = −0.84 to 0.01; figure 1*c*). Subjects performed significantly above chance in both *aligned* (Wilcoxon test: *W* = 78, *p* = 0.002) and *misaligned* conditions (Wilcoxon test: *W* = 75.5, *p* = 0.048; see electronic supplementary material for individual performances). These results are in contrast to Christie *et al*.'s [1] findings—chimpanzees and bonobos only performed well in the *aligned* condition. However, the *misaligned* condition differs between both studies: whereas in Christie *et al*.’ [1] study subjects had to *learn* a non-parallel mapping strategy (e.g. seeing food being hidden in the top position should lead to search for the reward in the bottom position of the searching array), in the present study, bees chose between two strips that matched either the relative or absolute position of the baited strip. Additionally, bees' choices were never rewarded. Thus, these differences in methodology could explain why bees favoured relational over positional cues.

Subjects were significantly above chance in the first trial of the *aligned* condition (binomial test, *p* = 0.035) but not of the *misaligned* condition (binomial test, *p* = 1). The advantage of the parallel alignment (i.e. *aligned* condition) compared to the oblique alignment (i.e. *misaligned* condition) was only evident in the first trial. There were no differences in performance between the first six and the last six trials in either of the conditions (*aligned*: *W* = 29.5, *p* = 0.878; *misaligned*: *W* = 19.5, *p* = 0.440)—suggesting that motivation did not affect subjects’ performances.

These results indicated that bumblebees, like primates, spontaneously succeeded at a spatial mapping task. Importantly, previous research has shown that when relational similarity competes with object similarity, young children [1] prefer object over relational match. This is not the case for other vertebrates (e.g. primates: [1]). In fact, it has been argued that a greater focus on object similarity in humans could be contributing to the analogical gap between humans and other animals (e.g. [16]). Bees rely on multimodal signals (e.g. colour and patterns of tactile surface structure) to learn floral patterns and improve their foraging effectiveness (e.g. [19,20]). As such, the effect of the object matches might be particularly obvious in bees. To test this possibility, object matches were introduced to the task in a new experiment.

## 3. Experiment 2: relational versus object similarity

**Figure 2.**
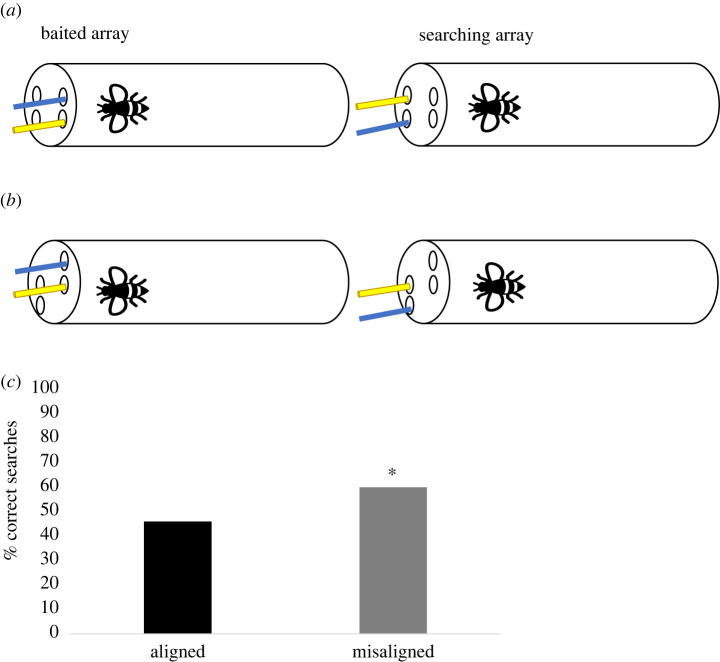
Experimental set-up of Experiment 2. Bees experience one of the stimuli baited on the (i) (E's perspective; baited array), and they had to search among the containers on the (ii) (searching array). In contrast with Experiment 1, the objects were now different. In both the aligned (*a*) and misaligned (*b*) conditions, the correct mapping between hide and search is the parallel spatial relations. Importantly, in the misaligned condition (*b*), the bottom object of the baited array spatially matches the top object of the search array. Thus, although the same objects were spatially aligned, a correct relational response would involve searching in the misaligned non-matching object (i.e. blue strip). It was predicted that if subjects attended to object matches over relational matches, they would struggle in these trials. Consequently, a worse performance in the *misaligned* condition compared to the *aligned* would be predicted; (*c*) represents the percentage of correct responses in the aligned and misaligned conditions (Experiment 2). Bees only searched in the correct location significantly above chance in the misaligned condition. *p < 0.05.

Similar to Experiment 1, in two conditions—*aligned* and *misaligned*—bees were presented with baited and searching arrays, but, in contrast with Experiment 1, the objects were now different (i.e. a blue strip and a yellow paper stick). The same objects were used for the baited and searching arrays, although they were distributed differently. Whereas in the baited array, the blue strip was at the top and the yellow paper stick was at the bottom, the objects' position was reversed in the searching array (figure 2*a,b*). Thus, in the *aligned* condition, object matches competed with the spatial relational rule (i.e. aligned searches). In the *misaligned* condition, the alignment of the object was oblique. Of particular importance were the trials in which the bottom object of the baited array was rewarded. This is because this bottom object matched the top object of the searching array (i.e. yellow stick). Thus, although the same objects were spatially aligned, a correct relational response would involve searching in the misaligned non-matching object (i.e. blue strip; figure 2*a,b*). It was predicted that if subjects were to focus on object matches over relational matches, they would struggle in these trials. Consequently, a worse performance in the *misaligned* condition compared to the *aligned* would be predicted.

### (a) Methods

#### (i) Subjects

The data were collected between August and September 2021 in Northumberland (UK). A total of 32 bees were captured; however, two of them completed less than six trials and were not included in the analyses. The final sample was 30 bees of the following species: *Bombus pascuorum* (*n* = 23), *Bombus terrestris* (*n* = 4) and *Bombus rupestris* (*n* = 3). Sex was visually identified (females = 13; males = 15 and two could not be clearly identified).

#### (ii) Apparatus

The same tubes as in Experiment 1 were used. Blue strips of paper (3 × 0.20 cm) and yellow paper lollipop sticks (3 × 0.32 cm) were used as stimuli (figure 2*a,b*). The objects were inserted in Playdoh to introduce them in the tube simultaneously.

#### (iii) Procedure

As before, bees were left in the tube on average for 2 h prior to testing. The same procedure as in Experiment 1 was followed for the *aligned* and *misaligned* conditions. Bees received a total of 12 trials, no training was provided prior to these trials and bees’ choices were never rewarded.

#### (iv) Analyses

The same analyses as in Experiment 1 were conducted. In both *aligned* and *misaligned* conditions, spatial matches were considered as correct.

### (b) Results and discussion

Subjects’ performance was affected by condition (estimate s.d. = 0.523, *z* = 2.350, *p* = 0.018, 95% CI = 0.07 to 0.98)—with bees performing better in the *misaligned* condition than in the *aligned* condition (figure 2*c*). In fact, bees performed significantly above chance in the *misaligned* condition (Wilcoxon test: *W* = 74.5, *p* = 0.045) but not in the *aligned* condition (Wilcoxon test: *W* = 24, *p* = 0.293). Importantly, in the *misaligned* condition, bees' performances did not differ between the bottom (i.e. incorrect object match competes with correct spatial match) and top trials (Wilcoxon test: *W* = 91.5, *p* = 0.078)—suggesting that bees’ searches were not affected by the competing object match.

As in Experiment 1, there were no differences in performance between the first six and the last six trials in either of the conditions (*aligned*: *W* = 44.5, *p* = 0.323; *misaligned*: *W* = 59, *p* = 0.706)—implying that change in motivation did not play a role in bees' choices.

In the present paradigm, bees did not seem to focus on object matches. This finding is supported by their performance in the *misaligned* condition. However, bees' difficulty in the *aligned* condition suggests that the presence of object matches might have affected their responses—recall that in Experiment 1 bees performed significantly above chance in the *aligned* condition. In fact, individual performance (electronic supplementary material) indicates that whereas 40% of the subjects usually used a relational strategy, 40% frequently relied on an object matching strategy. Thus, two competing strategies seem to be at play in the *aligned* condition. It is possible that the lack of objects next to the top hole in the baited array and to the bottom hole in the searching array in the *misaligned* condition might have facilitated the use of the relational mapping strategy.

## 4. General discussion

The present findings indicate that wild bumblebees can *spontaneously* attend to relational similarity. Importantly, when object matches competed with relational matches, bees tended to focus on relations more than on objects.

Studies have shown that bees can learn to abstract relational representations (i.e. ‘sameness’) in the context of colours, smells, sizes and quantities (e.g. [5,8–11,21]). The results presented here extend previous findings by showing that bees spontaneously prefer relational similarity over object similarity. These results are along the lines of previously reported findings in non-human primates (e.g. [1,7]). Evidently, a preference for relational over object matches does not mean that bees cannot make object matches—in fact, some bees in Experiment 2 did show a preference for object matches. Importantly, bees' lifestyles incorporate effective learning and memory capabilities with complex navigation skills as well as flexible visual system for pattern recognition [22]—which surely facilitates object recognition and object matching. Also, previous evidence from DMTS indicates that bees match objects [5]. Thus, it is possible that the nature of the paradigm used here—i.e. number of trials and/or responses not being rewarded—affected bees’ performances. One would expect that if they had been given more trials and their searches had been rewarded, they might have been able to consistently exploit object matches.

The absence of object bias reported here extends previous findings in vertebrates (e.g. chimpanzees) to invertebrates (i.e. bumblebees). It has been argued that it is young children's abilities to establish comparisons between similar objects that, over time, contribute to a more elaborated capacity to establish abstract relationships. Thus, these results are of great importance because they help confirm that the relational gap between humans and other animals is not due to animals' inability to identify relational similarities but to their preference for relational over object similarities [16].

In conclusion, the present results question the view that vertebrates, and in particular non-human primates, may be the only animals able to *spontaneously* attend to relations of similarity—wild bumblebees can also do so. Moreover, bumblebees, like non-human primates, also focused strongly on relations over objects—which suggests that their cognition might not be as concrete as previously argued. Studies like the one presented here indicate that social insects are useful models for understanding the evolution of cognition.

## Data Availability

The data are provided in the electronic supplementary material [23].

## References

[1] Christie S, Gentner D, Call J, Haun DBM. 2016 Sensitivity to relational similarity and object similarity in apes and children. Curr. Biol. **26**, 531-535. (10.1016/j.cub.2015.12.054) 26853364

[2] Haun DBM, Call J. 2009 Great apes' capacities to recognize relational similarity. Cognition **110**, 147-159. (10.1016/j.cognition.2008.10.012) 19111286

[3] Penn DC, Holyoak KJ, Povinelli DJ. 2008 Darwin's mistake: explaining the discontinuity between human and nonhuman minds. Behav. Brain Sci. **31**, 109-130. (10.1017/S0140525X08003543) 18479531

[4] Smirnova A, Zorina Z, Obozova T, Wasserman E. 2015 Crows spontaneously exhibit analogical reasoning. Curr. Biol. **25**, 256-260. (10.1016/j.cub.2014.11.063) 25532894

[5] Giurfa M, Zhang S, Jenett A, Srinivasan MV. 2001 The concepts of ‘sameness’ and ‘difference’ in an insect. Nature **410**, 930-933. (10.1038/35073582) 11309617

[6] Fagot J, Wasserman EA, Young ME. 2001 Discriminating the relation between relations: the role of entropy in abstract conceptualization by baboons (*Papio papio*) and humans (*Homo sapiens*). J. Exp. Psychol. Anim. Behav. Process. **27**, 316-328. (10.1037/0097-7403.27.4.316) 11676083

[7] Hribar A, Haun DBM, Call J. 2011 Great apes’ strategies to map spatial relations. Anim. Cogn. **14**, 511-523. (10.1007/s10071-011-0385-6) 21359655

[8] Avarguès-Weber A, Finke V, Nagy M, Szabó T, d'Amaro D, Dyer AG, Fiser J. 2020 Different mechanisms underlie implicit visual statistical learning in honey bees and humans. Proc. Natl Acad. Sci. USA **117**, 25 923-25 934. (10.1073/pnas.1919387117) PMC7568273 32989162

[9] Howard SR, Avarguès-Weber A, Garcia JE, Greentree AD, Dyer AG. 2018 Numerical ordering of zero in honey bees. Science **360**, 1124-1126. (10.1126/science.aar4975) 29880690

[10] Howard SR, Avarguès-Weber A, Garcia J, Dyer AG. 2017 Free-flying honeybees extrapolate relational size rules to sort successively visited artificial flowers in a realistic foraging situation. Anim. Cogn. **20**, 627-638. (10.1007/s10071-017-1086-6) 28374206

[11] Brown M, Sayde J. 2013 Same/different discrimination by bumblebee colonies. Anim. Cogn. **16**, 117-125. (10.1007/s10071-012-0557-z) 22945434

[12] Chittka L. 2017 Bee cognition. Curr. Biol. **27**, R1049-R1053. (10.1016/j.cub.2017.08.008) 29017035

[13] Loukola OJ, Solvi C, Coscos L, Chittka L. 2017 Bumblebees show cognitive flexibility by improving on an observed complex behavior. Science **355**, 833-836. (10.1126/science.aag2360) 28232576

[14] Heinrich B. 1979 Majoring and minoring by foraging bumblebees, *Bombus vagance*: an experimental study. Ecology **60**, 245-255. (10.2307/1937652)

[15] Ohashi K, Leslie A, Thomson JD. 2008 Trapline foraging by bumble bees: V. Effects of experience and priority on competitive performance. Behav. Ecol. **19**, 936-948. (10.1093/beheco/arn048)

[16] Christie S. 2021 Learning sameness: object and relational similarity across species. Curr. Opin. Behav. Sci. **37**, 41-46. (10.1016/j.cobeha.2020.06.010)

[17] Muth F, Cooper TR, Bonilla RF, Leonard AS. 2017 A novel protocol for studying bee cognition in the wild. Methods Ecol. Evol. **9**, 78-87. (10.1111/2041-210X.12852)

[18] Bolker BM, Brooks ME, Clark CJ, Geange SW, Poulsen JR, Stevens MH, White JS. 2009 Generalized linear mixed models: a practical guide for ecology and evolution. Trends Ecol. Evol. **24**, 127-135. (10.1016/j.tree.2008.10.008) 19185386

[19] Kulahci IG, Dornhaus A, Papaj DR. 2008 Multimodal signals enhance decision making in foraging bumble-bees. Proc. R. Soc. B **275**, 797-802. (10.1098/rspb.2007.1176) PMC2596894 18198150

[20] Nityananda V, Chittka L. 2015 Modality-specific attention in foraging bumblebees. R. Soc. Open Sci. **2**, 150324. (10.1098/rsos.150324) 26587245 PMC4632517

[21] Giurfa M. 2021 Learning of sameness/difference relationships by honey bees: performance, strategies and ecological context. Curr. Opin. Behav. Sci. **37**, 1-6. (10.1016/j.cobeha.2020.05.008) 35083374 PMC8772047

[22] Chittka L, Jensen K. 2011 Animal cognition: concepts from apes to bees. Curr. Biol. **21**, R116-R119. (10.1016/j.cub.2010.12.045) 21300275

[23] Martin-Ordas G. 2022 Data from: Spontaneous relational and object similarity in wild bumblebees. *FigShare*. (10.6084/m9.figshare.c.6152133) PMC9428533 36043304

